# Keeping memories alive

**DOI:** 10.3389/fimmu.2013.00021

**Published:** 2013-02-01

**Authors:** Gabrielle T. Belz, Erika Cretney

**Affiliations:** ^1^Division of Molecular Immunology, Walter and Eliza Hall Institute of Medical ResearchMelbourne, VIC, Australia; ^2^Department of Medical Biology, The University of MelbourneMelbourne, VIC, Australia

Memory T cells confer immune protection against a diverse range of pathogens. The quantity and quality of responding memory T cells depends on a number of factors including cytokines, recognition of pathogen-derived MHC complexes, and costimulatory molecules. Integration of these signals ensures that T cell responses are tightly regulated. Delineating the mechanisms that regulate the differentiation, establishment, and maintenance of memory T cells is fundamental to life-long immune protection and for engineering of effective T cell-based vaccines.

Despite extensive research dissecting the features of T cell memory, many aspects of this process remain incompletely understood. This is in part due to a realization that integral to protective memory is the existence of multiple T cell subsets with diverse distributions to lymphoid, mucosal, and non-lymphoid sites. While there is a consensus that T cell memory is essential, which cells provide effective memory in different infections and at different anatomical sites evokes considerable debate.

This volume brings together 10 articles that are intended to summarize the current thinking on the development of immunological memory and to highlight important areas of investigation for the future in teasing apart the ability of the immune system to preserve the knowledge of a previously encountered antigen or pathogen and to use this to vigorously defend against a second or subsequent infection.

The first article (Hamilton and Jameson, 2012) introduces the feature of CD8^+^ T cell memory and the cellular and transcriptional factors that influence memory T cell formation. This review introduces new subsets that have begun to populate the memory T cell landscape such as the self-renewing “memory stem cells” and effector-like memory cells and raises the important question of which cells within the memory compartment (effector, effector-like, and central memory) actually provide immune protection? In general, the broad picture painted of an immune response arises from viewing outcomes at a population level. However, as shown by Buchholz et al. (2013), drilling down on the decision processes of a single cell is now technically feasible, offering unparalleled opportunity to determine how the population relates to the choices of individual cells and the ability to search deeply for putative “memory stem cells” and how they contribute to shaping the memory compartment.

The next article by Gebhardt and Mackay (2012) discusses in depth the role of non-lymphoid tissue-resident memory CD8^+^ T cells. Although experimental models often tackle questions of T cell memory using systemic models of infection, vast exposure of organisms occurs through contact of mucosal and cutaneous tissues, placing how the body deals with localized infections in the peripheral tissues as a major and critical arm of protective immunity. Evolving concepts of memory T cell formation that include the subpopulation of memory T cells that express the integrin CD103 are described, as are human CD8^+^CD103^+^ memory T cells that share a multitude of characteristics with tissue-resident memory T cells described in mice.

This leads on to a cluster of three articles that address how differentiation affects survival and persistence of memory T cells and the impact of these features to vaccination approaches (Kedzierska et al., 2012; Kurtulus et al., 2012; Vasconcelos et al., 2012). The traditional view of T cell memory has revolved around the development of a small residual stable memory pool (~5% of expanded cells) of T cells following massive expansion and extensive contraction or death of many pathogen-specific cells in response to infection. This pattern of development promoted the idea of a linear model of T cell development that has been widely accepted. Nevertheless, other studies provided non-concordant insights suggesting that alternate models might account for the fate decisions undertaken by T cells. Indeed it is now clear that the signals received by T cells early in infections are likely to be highly instructive in dictating the more immediate outcome of a response. These early cues also appear to imprint the T cells with a program that ensures their longevity through the establishment of a cell survival program. This contrasts with the diminished response elicited from “old” naïve T cells when confronted by a pathogen in an immune response and provides important insights to how we might shape the immune response at the point of initial challenge through vaccination approaches.

Co-ordinate regulation of surface molecules and the internal machinery fine tune the transferral of external signals to the molecular apparatus controlling T cell fate decisions. This is initiated by engagement of the T cell antigen receptor (TCR). Using super high-resolution microscopy, Rossy et al. (2012) discuss new spatiotemporal models that regulate TCR organization and the impact of these new concepts on our understanding of T cell activation and differentiation. TCR activation ultimately induces a signaling cascade that results in the induction of a transcriptional program that results in terminal differentiation of T cells (Russ et al., 2012; Kim and Suresh, 2013). Kim and Suresh (2013) discuss in detail the role of phosphatidylinositol 3-kinase (PI3K)/Akt/mTor signaling pathway which is positioned to coordinate the convergence of TCR and costimulatory signals and influence CD8^+^ T cell effector/memory fate decisions principally by regulating cellular metabolism Superimposed on these activatory programs are epigenetic changes that fine-tune gene transcription or silencing (Russ et al., 2012).

The volume concludes with two articles that examine the development of T cell memory following malaria (Krzych et al., 2012) and helminth (Harvie et al., in review) infection. Using the *P. berghei* γ-spz mouse model, Krzych et al. (2012) have identified two populations of intrahepatic memory CD8^+^ T cells: IFN-γ-producing CD8^+^ T effector/effector memory cells and CD8^+^ T central memory cells. They follow with a model that proposes that liver T central memory cells are maintained by IL-15, and that CD8^+^ T effector/effector memory cells are “conscripted” from this population to prevent re-infection. In contrast to *P. berghei* infection, the adaptive immune response to parasitic roundworm infection is mediated mainly by the effector cytokines produced by CD4^+^ T cells. Harvie et al. (in review) discuss a number of interesting features of this response including the capacity to generate long-term local protection, particularly in the lung—a site where priming may be sufficient to confer protection.

In summary, these articles present a range of aspects of immune memory formation that are currently under investigation. They bring together current knowledge and models that transport us from the initial signaling events at the cell surface to the internal workings that guide the fate decisions of memory T cells in dealing with different types of infections. Furthermore, they highlight the challenges that still confront the field in unraveling what defines memory and how these characteristics are optimized during infection or designing strategies for vaccination.
